# Impact of the COVID-19 Infection on Head and Neck Cancer Treatment During Hospitalization

**DOI:** 10.7759/cureus.60578

**Published:** 2024-05-19

**Authors:** Ryo Kawaura, Hirofumi Shibata, Hiroshi Okuda, Kosuke Terazawa, Takenori Ogawa

**Affiliations:** 1 Otolaryngology - Head and Neck Surgery, Gifu University Graduate School of Medicine, Gifu, JPN

**Keywords:** hospitalization, chemoradiotherapy, radiotherapy, head and neck cancer, covid-19

## Abstract

Objective

The after-effects of coronavirus disease 2019 (COVID-19) infection are still unknown; therefore, we investigate the possibility that COVID-19 may interrupt cancer treatment and impact prognosis.

Methods

We examined the characteristics, prognosis, and changes in treatment options before and after COVID-19 in 11 head and neck cancer patients who were infected with COVID-19 after admission for cancer treatment at Gifu University Hospital. These patients were compared to 110 patients unaffected by COVID-19 during the same period. To identify the effects of COVID-19 on the cancer treatment sequence, we examined the rates of overall survival, progression-free survival, and completion, as well as radiation dosage in radiotherapy and cisplatin dosage in chemoradiotherapy.

Results

All 11 patients with COVID-19 had their planned cancer treatment postponed or interrupted. There was no significant difference in overall or progression-free survival compared to patients without COVID-19. Notably, only 3/6 of the COVID-19-affected patients completed radiotherapy compared to 42/46 unaffected patients. The ratio of actual radiation dose to planned dose was significantly impaired in COVID-19 affected patients group (98.3% vs. 88.6%). Cisplatin dosage in chemoradiation was not significantly different in either the radical (100 mg/m^2^, every three weeks) or adjuvant (40 mg/m^2^, every one week) treatment groups.

Conclusion

COVID-19 infection in head and neck cancer patients had no apparent impact on cancer prognosis. However, when restricted to irradiation, the treatment completion rate and the ratio of planned to actual dose decreased significantly, underscoring the impact of COVID-19 infection on cancer treatment. The difference in irradiation may affect the success of patients’ treatment going forward, and it should be explored whether irradiation can be continued without delay.

## Introduction

Although coronavirus disease 2019 (COVID-19) has not been eliminated, the world is returning to pre-pandemic life, with the World Health Organization declaring that the “international public health emergency” concerning the novel coronavirus infection will conclude in May 2023 [1]. In Japan, the classification of COVID-19 under the Infectious Diseases Control Law [2] has concurrently transitioned from “category 2 equivalent,” which includes tuberculosis and severe acute respiratory syndrome (SARS) and must be monitored at medical facilities nationwide, to “category 5,” which includes seasonal influenza and is subject to fixed-point surveillance at designated medical facilities. However, the impact of COVID-19 on daily practice is still significant [3]. Past reports [4-7] indicate that cancer was one of the risk factors influencing the severity of COVID-19 infection in the early pandemic period.

Several reports have addressed the impact of COVID-19 on cancer treatment. Mitsui et al. [8] reported that serum interleukin (IL)-10 levels were higher in severe cases of COVID-19 before the onset of severe disease. Serum IL-10 suppresses the activity of cytotoxic T cells as an inhibitory cytokine and is also involved in tumor immune escape. An epigenetic mechanism has also been evaluated in which NSP1, one of the proteins encoded by the severe acute respiratory syndrome coronavirus 2 (SARS-CoV-2) virus, promotes methylation of histones through histone 3 lysine 9 dimethylation (H3K9me2) expression, thereby repressing host immune-related genes [9]. Based on these findings, we hypothesize that COVID-19-induced immunosuppression during cancer treatment may influence tumor immunity and impact head and neck cancer prognosis.

To date, there is only one case report of esophageal cancer treatment [10] and several reports on the impact of COVID-19 on overall medical treatment [11-12]. In the head and neck region, there may be a risk of upper respiratory tract symptoms due to COVID-19 infection and associated deterioration of the general condition. In addition to the early reports of the COVID-19 pandemic [13], Koizumi et al. [14] reported on the impact of the COVID-19 pandemic on the number of otolaryngological surgeries, and Nadarajan et al. [15] reported on the association between surgical complications and COVID-19. Nevertheless, to the best of our knowledge, there are no reports documenting the influence of COVID-19 on managing head and neck malignancies in the inpatient setting (as per a PubMed title search employing the query “COVID-19 head and neck cancer hospitalization”). Within our medical unit, 10 head and neck cancer inpatients encountered COVID-19 infections during the autumn of 2022. Thus, in this study, we examine the impact of COVID-19 infection on head and neck cancer treatment.

## Materials and methods

Study of COVID-19-affected cases

A total of 17 cases at Gifu University Hospital were identified from electronic medical records as meeting the following criteria: (1) admission after the onset of the COVID-19 pandemic in Japan (April 2020 to December 2022), (2) admission to the Department of Otorhinolaryngology-Head and Neck Surgery, and (3) documentation of the disease names “cancer” and “COVID-19” in the electronic medical record system. From this cohort, 12 individuals were diagnosed with COVID-19 during head and neck cancer treatment post-admission. A comprehensive retrospective investigation was conducted, encompassing cancer type, clinical stage, age, gender, duration of hospitalization, COVID-19 treatment, isolation duration, original and realized cancer treatment plans (with or without modifications), radiation dosage, and outcomes. The clinical staging adhered to the Union for International Cancer Control (UICC) Tumor, Node, Metastasis (TNM) Classification of Malignant Tumors, 8th edition [16], and underwent scrutiny by the in-house cancer board.

Comparison with COVID-19 non-infected cases during the same period

A total of 141 novel cases of head and neck cancer were admitted to our institution during the temporal span of the COVID-19 pandemic (April 2022 to March 2023). Of these, 31 patients were omitted for the following reasons: (1) 11 admissions for diagnostic purposes exclusively, devoid of therapeutic interventions, or for purposes unrelated to cancer treatment were eliminated; (2) one brief admission for chemotherapy, encompassing the initiation of new monotherapy involving immune checkpoint inhibitors, was categorized as an outpatient scenario; and (3) 19 instances of thyroid cancer were excluded due to significant prognostic disparities from other head and neck malignancies. As a result, 110 patients with head and neck carcinomas were included and compared to the COVID-19-affected cases above. The primary endpoint was overall survival (OS) and progression-free survival (PFS), and the secondary endpoint was treatment completion rates, radiation doses among radiotherapy patients, and total cisplatin (CDDP) doses in CDDP combined chemoradiotherapy (CCRT). The reason for focusing on radiotherapy and CDDP combined CCRT, either radical (100 mg/m^2^ every three weeks) or adjuvant (40 mg/m^2^ every one week), was that radiotherapy is basically a one-time treatment for the same site. The comparisons are simpler and clearer than for other chemotherapy treatments, which have many variations in regimens and doses. Because the number of patients with COVID-19 was small, radical irradiation, postoperative irradiation, and palliative irradiation were considered together for both the COVID-19-affected and unaffected groups. Clinical staging adhered to the UICC TNM classification [16], as described above. All individuals underwent polymerase chain reaction (PCR) testing (utilizing nasopharyngeal or salivary specimens) to determine SARS-CoV-2 infection and were confirmed negative at the time of admission. Mac Mini, operating system 14.2.1 (Apple Inc., USA) and R software, version 4.3.2 (www.r-project.org) were used for statistical analysis, and the Kaplan-Meier method was used for OS and PFS with the log-rank test. Fisher’s exact test was used to compare treatment completion rates for radiation therapy, and the Wilcoxon rank sum test was used to compare irradiation doses and total CDDP dose, with *p*<0.05 considered statistically significant for both tests. Approval for this study was granted by our hospital’s Clinical Research Ethics Review Committee (review number 2023-277).

## Results

Details of COVID-19-affected cases

Components of the 12 Cases Studied

The average age of the 12 patients, comprising 11 males and one female, was 68.42 years, with a median age of 73 years. The cancer types were composed of two cases of laryngeal cancer, two cases of hypopharyngeal cancer, two cases of oropharyngeal cancer, one case of nasopharyngeal cancer, two cases of maxillary cancer, one case of tongue cancer, one case of gingival cancer, and one case of olfactory neuroblastoma. Most cases were clinical stage IV (eight out of 11 cases, excluding one case of olfactory neuroblastoma). Of these, one male case of maxillary carcinoma was excluded based on the criteria mentioned above because the patient was readmitted for skin valve reconstruction after radical treatment. The remaining 11 cases are discussed in the next section. 

Details of 11 Cases Before and After COVID-19

Cancer treatment planned before COVID-19 was chemoradiotherapy in seven cases, chemotherapy in three cases, and surgery in one case. Regarding the history of vaccination with the novel coronavirus vaccine, except for one case that could not be confirmed, nine of the 10 patients had received multiple doses of the vaccine.

After COVID-19 infection, no cases were classified as severe, requiring ventilatory management or admission to the intensive care unit; two cases were moderate II, requiring oxygen administration; and the remaining nine cases were mild, requiring isolation only. After treatment with induction chemotherapy, molnupiravir was used in five cases, and nirmatrelvir/ritonavir was used in three cases to treat the novel coronavirus infection; no deaths occurred due to COVID-19, but two of the patients with moderate II disease and one patient with mild disease subsequently died of their primary illness. COVID-19 forced all patients to postpone, interrupt, or change their cancer treatment. Radiotherapy was completed before the planned dose was reached in three of the six patients undergoing treatment (one patient was ill before the start of irradiation and subsequently completed the planned dose).

Comparison with COVID-19 uninfected cases

Details of Affected and Unaffected Cases

The mean age of the group not affected by COVID-19 was 71.56 years, with a median age of 72 years. Gender distribution consisted of 81 males and 29 females. Most of the unaffected group also exhibited stage III/IV advanced cancer (67 out of 110 cases or 61.0%) (Table 1).

**Table 1:** Comparison of COVID-19-affected and unaffected cases CRT=chemoradiotherapy, ICI=immune checkpoint inhibitor.

		COVID (-)	COVID (+)			COVID (-)	COVID (+)			COVID (-)	COVID (+)
Age	average	67.72	71.56	cStage	I	10	0	Purpose	radical	62	4
	mean	71	72		II	24	1		adjuvant	1	3
Sex	male	81	10		III	14	2		salvage	28	2
	female	29	1		IV	53	7		palliative	19	2
Cancer	oral	27	3								
	nasopharynx	1	1	Main treatment	operation	60	1	Recurrence	no	72	8
	oropharynx	18	1		radiation	7	0		yes	38	3
	hypopharynx	22	2		chemotherapy*	43	10				
	larynx	10	2		*(includes additional treatments)			Prognosis	alive	89	8
	nasal	7	0		CRT	15 (24)	7		dead	21	3
	maxillary	4	1		solo	17 (18)	2				
	salivary	13	0		ICI	11 (14)	1				
	other	8	1					Total		110	11

Overall Survival and Progression-Free Survival

**Figure 1:**
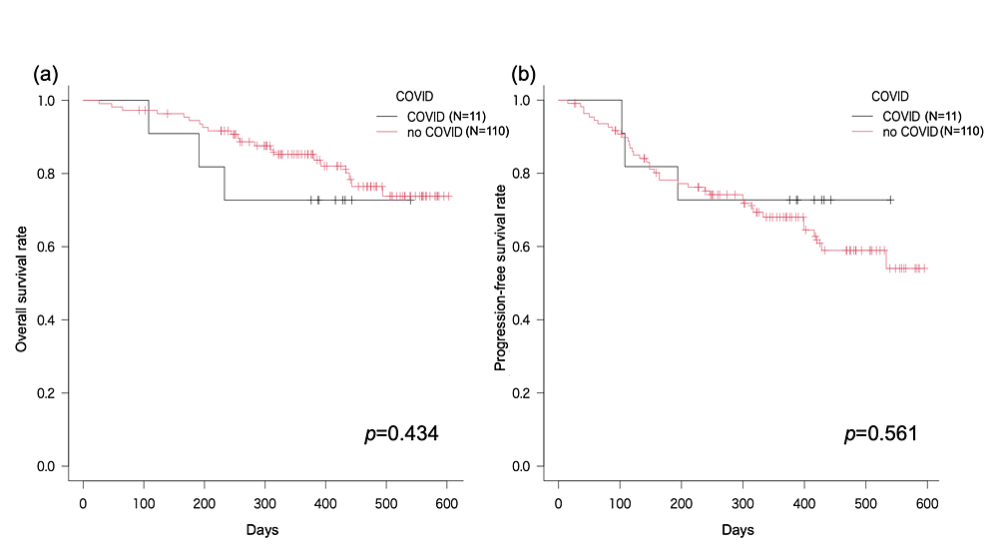
OS and PFS of patients with and without COVID-19 (a) Overall survival (OS) and (b) progression-free survival (PFS) of patients with and without COVID-19. There was no significant difference in overall or progression-free survival based on COVID-19 status.

The median follow-up duration was 13.0 months in the COVID-19 group and 12.6 months in the non-COVID-19 group, displaying no statistically significant disparities in OS (*p*=0.434) or PFS (*p*=0.561) based on COVID-19 status. Given that neither OS nor PFS fell below the 50% threshold for survival and progression-free relapse during the analytical period, median values were not computed (Figure 1).

Radiotherapy Completion Rate and Dose Comparison

**Figure 2:**
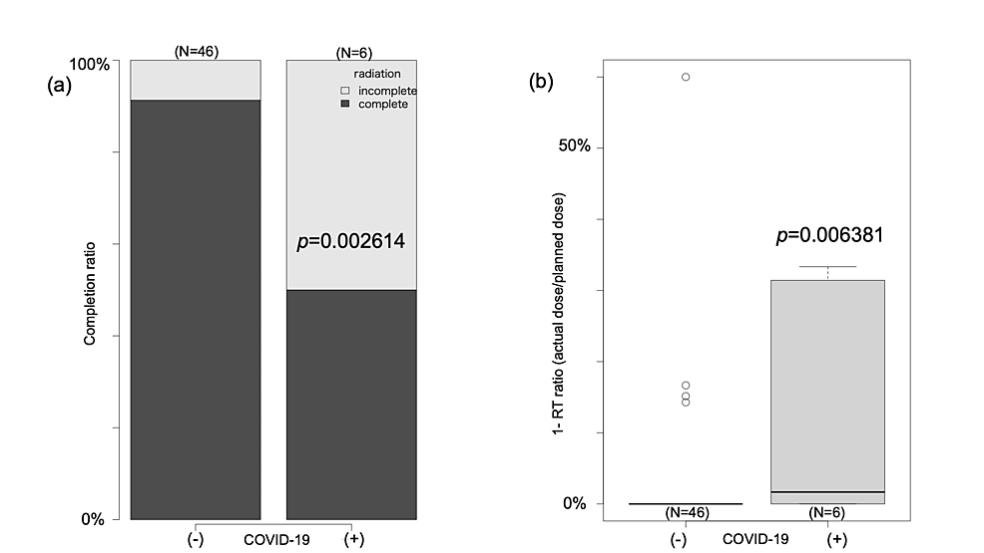
Ratio of radiation in irradiation cases (a) Irradiation completion rates by the presence of COVID-19 infection. The vertical axis indicates the number of cases. (b) Dose ratios by COVID-19 infection. The vertical axis indicates the ratio of the actual irradiation dose to the planned dose. Both the completion rate and dose ratio exhibited statistically significant distinctions.

Within the non-COVID-19 group, 42 out of 46 patients (91.3%) successfully attained the prescribed dosage, while only three out of six patients (50.0%) in the COVID-19-affected group accomplished the same. The ratio of the actual administered dose to the intended dose was 98.3% in the unaffected group, in contrast to 88.6% in the affected group. Both the completion rate (*p*=0.0261) and dose ratio (*p*=0.006381) exhibited statistically significant distinctions (Figure 2).

*Comparison of Total Cisplatin* D*ose in Cisplatin Combination Chemoradiotherapy*

**Figure 3:**
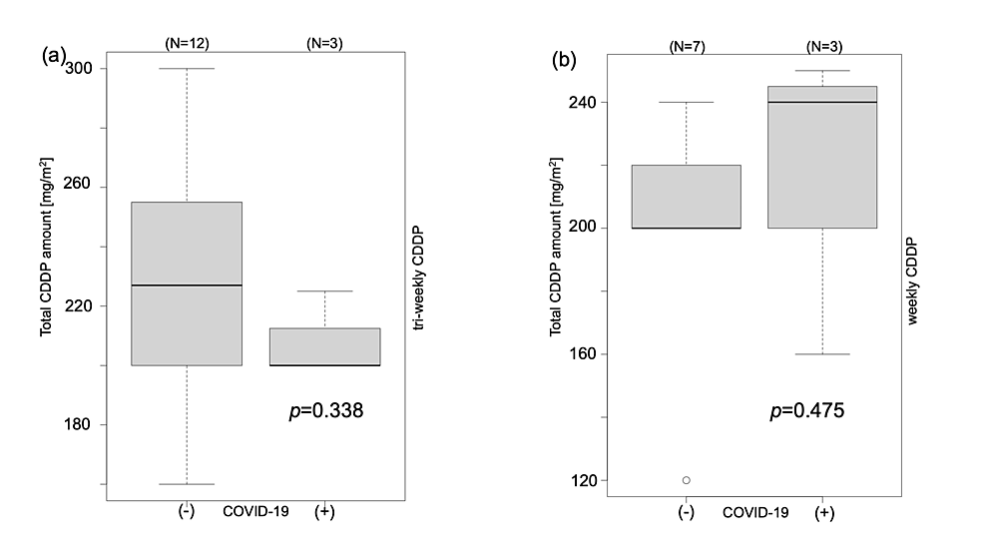
Total cisplatin (CDDP) dose by COVID-19 disease Cisplatin (CDDP) dosage for concurrent chemoradiotherapy (a: definitive treatment; CDDP 100 mg/m^2^ every three weeks) or postoperative treatment (b: adjuvant treatment; CDDP 40 mg/m^2^ weekly) does not indicate a statistical difference.

The cumulative CDDP dosage for concurrent chemoradiotherapy (CDDP 100 mg/m^2^ every three weeks) as a therapeutic modality exhibited a median of 227 mg/m^2^ (IQR: 200-252.5) among 12 individuals in the cohort unaffected by COVID-19, as opposed to a median of 200 mg/m^2^ (IQR: 200-212.5) in the three subjects within the COVID-19-affected group. This disparity, though not statistically significant (*p*=0.338), merits attention. Similarly, for postoperative treatment (CDDP 40 mg/m^2^ weekly), no statistically significant discrepancy was observed (*p*=0.475), with a median of 200 mg/m^2^ (IQR: 200-220) in the seven patients unimpacted by COVID-19, compared to 240 mg/m^2^ (IQR: 200-245) in the three affected patients (Figure 3).

## Discussion

In all 11 cases, the original cancer treatments (chemotherapy, irradiation, or postoperative procedures) were postponed or interrupted due to COVID-19 infection. The seven patients initially treated maintained complete remission of their cancer. Of the three patients who succumbed, two were categorized with intermediate disease according to Japanese guidelines [17] concerning COVID-19. Further case accumulation is warranted to elucidate the potential impact of COVID-19. Subsequently, a comparison of COVID-19-affected and unaffected cases showed no statistically significant difference in OS or PFS due to COVID-19. This result may come from, at least in part, the prevalence of mild cases among COVID-19-affected patients.

Notably, for radiotherapy, significant differences were observed in the treatment completion rate and the ratio of actual irradiation dose to planned dose. Although the present results alone are insufficient to conclusively determine the impact on the prognosis of failing to complete radiotherapy, the effect of COVID-19 cannot be ignored, as it forced the suspension of unscheduled irradiation. The total CDDP dose was not significantly different in either the radical (100 mg/m^2^ every three weeks) or adjuvant (40 mg/m^2 ^every one week) treatment groups, and it should be noted that the last dose of CDDP had already been administered at the time of COVID-19 infection in some cases. According to the recommendation of the Japanese Society of Radiation Oncology, "the COVID-19-affected confirmed cases or pseudo-cases, even those in close contact, should be reviewed on a case-by-case basis, whether curative or palliative and necessary radiation therapy should be continued as much as possible and initiated without delay” [18]. In the chemoradiotherapy cases examined in this study, COVID-19 infection caused up to three weeks of irradiation interruption. It has been suggested that tumor tissue becomes resistant to radiotherapy due to accelerated repopulation after 28 days of irradiation, according to in vitro data on the biological response of squamous cell carcinoma of the head and neck to radiation [19]. In practice, it has also been reported that delay or interruption of radiotherapy in squamous cell carcinoma of the larynx [20-21] and nasopharynx [22] leads to accelerated repopulation, leading to an undesirable prognosis. While the combined CDDP dose was not significantly different, differences in irradiation dose may improve treatment efficacy going forward. Therefore, careful judgment is required to determine whether treatment can be resumed as soon as possible while preventing the spread of the infection to other patients.

There are several limitations of this study. First, a large proportion of cases were in the advanced stage, which may have different prognoses compared with early cases because advanced patients often show severe complications. Second, the low number of COVID-19 patients with various cancer subsites and different treatments makes it difficult to draw a clear conclusion. Although we avoided subdivisions in this study due to sample size concerns, it would be desirable to look at additional cases in the future to examine cancer treatment through a more detailed analysis. Furthermore, future follow-up of cases is also required to determine the impact of failure to complete radiotherapy, as this observation period is insufficient to assess the full impact.

## Conclusions

In our clinical encounters with COVID-19 infection among head and neck cancer patients receiving inpatient care, the impact of the virus on cancer prognosis remains nebulous. There were scattered cases in which COVID-19 forced the interruption or termination of originally planned treatment, and there was no difference in total CDDP dose between patients with or without COVID-19. On the other hand, there were significant differences in completion rates and the ratio of planned dose to actual dose in irradiation. The long-term prognostic impact of COVID-19 morbidity in cancer patients is still unclear. The interruption of cancer treatment due to the disease places a burden on healthcare providers and leads to unnecessary anxiety for patients. In the “post-pandemic” era, paying close attention to infection control measures will be essential.
